# Entomological risk of African tick-bite fever (*Rickettsia africae* infection) in Eswatini

**DOI:** 10.1371/journal.pntd.0010437

**Published:** 2022-05-16

**Authors:** Kimberly J. Ledger, Hanna Innocent, Sifiso M. Lukhele, Rayann Dorleans, Samantha M. Wisely

**Affiliations:** 1 Department of Wildlife Ecology and Conservation, University of Florida, Gainesville, Florida, United States of America; 2 Department of Biological Sciences, University of Eswatini, Kwaluseni, Eswatini; 3 Department of Biological Sciences, University of Cyprus, Nicosia, Cyprus; University of Liverpool, UNITED KINGDOM

## Abstract

**Background:**

*Rickettsia africae* is a tick-borne bacterium that causes African tick-bite fever (ATBF) in humans. In southern Africa, the tick *Amblyomma hebraeum* serves as the primary vector and reservoir for *R*. *africae* and transmits the bacterium during any life stage. Previous research has shown that even when malaria has been dramatically reduced, unexplained acute febrile illnesses persist and may be explained by the serological evidence of rickettsiae in humans.

**Methodology/Principal findings:**

We collected 12,711 questing *Amblyomma* larvae across multiple land use types in a savanna landscape in Eswatini. Our results show that host-seeking *Amblyomma* larvae are abundant across both space and time, with no significant difference in density by land use or season. We investigated the entomological risk (density of infected larvae) of ATBF from *A*. *hebraeum* larvae by testing over 1,600 individual larvae for the presence of *R*. *africae* using a novel multiplex qPCR assay. We found an infection prevalence of 64.9% (95% CI: 62.1–67.6%) with no land use type significantly impacting prevalence during the dry season of 2018. The mean density of infected larvae was 57.3 individuals per 100m^2^ (95% CI: 49–65 individuals per 100m^2^).

**Conclusions:**

Collectively, our results demonstrate *R*. *africae* infected *A*. *hebraeum* larvae, the most common tick species and life stage to bite humans in southern Africa, are ubiquitous in the savanna landscape of this region. Increased awareness of rickettsial diseases is warranted for policymakers, scientists, clinicians, and patients. Early detection of disease via increased clinician awareness and rapid diagnostics will improve patient outcomes for travelers and residents of this region.

## Author summary

African tick-bite fever (ATBF) is a widespread, endemic tick-borne rickettsiosis caused by the bacterium *Rickettsia africae*. ATBF is second only to malaria as the cause of acute febrile illness in travelers returning from sub-Saharan Africa, and *R*. *africae* seropositivity rate (which is indicative of past infection) is high in indigenous populations from rural areas. The primary vector of *R*. *africae* in southern Africa is the South African bont tick, *Amblyomma hebraeum*. This tick is a highly aggressive human biter during the larval life stage. In this study, we quantified the density of infected *A*. *hebraeum* larvae across the lowveld savanna in Eswatini. We found over half of tested *A*. *hebraeum* larvae were infected with *R*. *africae*, and the density of infected larvae can be high in multiple land uses, including wildlife preserves, ranches, and community grazing lands. This high likelihood of a bite by an infected *A*. *hebraeum* in the savanna of Eswatini suggests ATBF poses a significant risk to travelers and may contribute to unexplained acute febrile diseases in the region.

## Introduction

Rickettsial diseases are vector-borne bacterial infections that cause acute febrile illness, and they are among the most common emerging or re-emerging zoonoses globally [1]. Rickettsial diseases are prevalent in tropical and subtropical regions of the world and disproportionately affect those living in poverty with limited access to modern health care [2]. For decades, rickettsial diseases have been an overlooked cause of morbidity, mortality, and economic losses in marginalized populations [3]. Despite the increasing awareness of the importance of rickettsial diseases, there remains a lack of information on the prevalence of rickettsiae in vector species across all infective life stages.

African tick-bite fever (ATBF), caused by the bacterium *Rickettsia africae*, is a neglected rickettsial disease transmitted by ticks in sub-Saharan Africa. *Rickettsia africae* belongs to the spotted-fever group (SFG) *Rickettsia*, which comprises a diverse group of pathogenic bacteria that can cause spotted fevers in humans. *Rickettsia africae* is predominately transmitted by the ticks, *Amblyomma variegatum* and *Amblyomma hebraeum* [4]. Both tick species can maintain rickettsiae within the tick population by vertical transmission of the bacteria from mother to egg [5,6]. Therefore, the distribution of the tick vectors determines the distribution of the *R*. *africae* pathogen. The distribution of *A*. *variegatum* spans western, central, and eastern parts of sub-Saharan Africa and the West Indies, whereas *A*. *hebraeum* is only in southern Africa [7].

ATBF is a neglected disease that can cause illness in travelers and rural inhabitants bitten by ticks in endemic regions [4,8,9]. Rickettsial infections are second only to malaria as the cause of acute febrile illnesses among travelers to sub-Saharan Africa [8]. Reports of ATBF in local populations are limited [4,9]. Still, studies that measure the seroprevalence of past *Rickettsia* infection indicate rates can reach 60–90% across the geographic range (central Africa: [10,11]; western Africa: [12]; southern Africa: [13,14]). Living and working in rural settings where cattle and *Amblyomma* ticks are present is linked with a heightened risk of *R*. *africae* infection. Other outdoor activities associated with vector contact, such as game hunting, can also increase risk [15,16]. The contribution of rickettsioses to undiagnosed acute febrile illnesses remains unknown due to the lack of laboratory diagnostic tests in rural and developing regions and the low reliability of test results even when the test is available [17]. In an area where malaria, the most common diagnosis for febrile patients in low-resource health care settings [18,19], has been dramatically reduced, unexplained acute febrile illnesses persist and may be explained by the serological evidence of rickettsiae in humans [20].

*Amblyomma hebraeum* larvae pose a significant risk of parasitism to humans as they are abundant and aggressive. A study in South Africa found *A*. *hebraeum* larvae were five times more abundant in the vegetation, over two times more abundant on human clothing, and inflicted more bites to humans than any other tick species or life stage [21]. Fully engorged females can lay between 6 and 18 thousand eggs [22], resulting in thousands of larvae available to attach to hosts. Larvae are small and may go undetected on the human body, resulting in longer attachment times. This extended time may lead to an increased potential for pathogen transmission, as the probability of transmission increases with time of attachment [23].

Members of SFG *Rickettsia* are relatively unique in their efficient transovarial transmission (passage from the adult female through the ovaries to the unfed larvae of the next generation). *Amblyomma hebraeum* and *A*. *variegatum*, naturally infected with *R*. *africae*, can maintain the bacterium through trans-stadial and transovarial transmission for multiple generations [5,6] as the majority of egg masses from *R*. *africae*-infected *A*. *hebraeum* females contain at least one *R*. *africae* positive egg [24]. The combination of high transovarial rates in transmission studies and high prevalence of infected ticks of all life stages from field collections has led to the conclusion that *R*. *africae* may be an endosymbiont of these tick vectors [25].

Previous studies on rickettsiae in southern African ticks have documented the presence of *R*. *africae* in *A*. *hebraeum* adults and nymphs [26,27]. *Rickettsia africae* infection in *A*. *hebraeum* is high, typically ranging from 60–80% [26–30]. Previous studies testing for *R*. *africae* in any region have either not included larvae [31,32] or failed to identify *Amblyomma* larvae to the species level [30,33]. Given that several species of *Amblyomma* ticks co-occur with *A*. *hebraeum* [34] and *Amblyomma* species can differ in *R*. *africae* prevalence [33,35], differentiating *Amblyomma* larvae to species level before pathogen analysis is essential for accurate estimates of infection prevalence.

This study investigated the entomological risk [36] of ATBF from *A*. *hebraeum* larvae across multiple land use types in a savanna landscape. We estimated entomological risk as the density of infected larvae (DIL) or probability of an infected *A*. *hebraeum* bite in multiple rural land use types in Eswatini. As *A*. *hebraeum* larvae are small, abundant, and aggressive ticks that can spread ATBF to humans, assessment of pathogen prevalence is a crucial step towards understanding the risk of rickettsial diseases in endemic areas. The entomological risk may differ by land use because microhabitat and host community composition, both of which are influenced by land use [37], can impact larval tick survival, development, and activity [38]. Identifying hazards across multiple land uses will aid in designing disease prevention measures through vector control and education awareness programs, as people’s interaction with the landscape can differ due to their occupation, recreational activities, and economic status.

## Methods

### Study area and sampling design

We conducted this study in the lowveld region of Eswatini. This region experiences mild, dry winters (8–26°C; 0-50mm) and hot, wet summers (15–33°C; 200-500mm) [39–41]. To quantify entomological risk over space, we selected 26 sites representing four common land uses of lowveld savanna grasslands in Eswatini (wildlife conservation reserves, cattle ranches, conservation ranches that maintain a mixture of cattle and game animals, and community Swazi Nation Lands near rural settlements often used for livestock grazing). We sampled all 26 sites during the dry season of 2018. All life stages of ticks were abundant in savanna grasslands, but not in agricultural areas, during the dry season [27,38]. To quantify entomological risk over time, we also sampled a wildlife conservation reserve, Mbuluzi Nature Reserve, five times over two years. We sampled two or three plots per site, where each site included 2 to 6 100-meter transects of drag sampling. We used a tape measure to mark straight transects and maintain 10 meters between transects within a plot. We accounted for differences in sampling effort per site in our density estimates. We selected the temporal sampling intervals to represent the dry and wet seasons in Eswatini.

We obtained research permits to collect ticks inside wildlife conservation reserves from the Eswatini National Trust Commission. We received verbal permission from landowners to collect ticks on government-owned cattle ranches, conservation ranches, and community Swazi Nation Lands. Permission to import dead, unengorged ticks to the United States was granted by the United States Department of Agriculture, the United States Centers for Disease Control and Prevention, and the United States Fish and Wildlife Service.

Our tick sampling technique has been previously described [27]. In short, to mimic how humans may encounter host-seeking ticks, we sampled ticks by dragging a 1m^2^ white flannel cloth across the vegetation allowing ticks to attach to the cloth as it passed. We inspected the cloth every 10 meters and spent a maximum of two minutes transferring ticks directly into 90% molecular-grade ethanol. Samples from unique transects and sampling sessions were collected and stored in individually labeled vials. We established a maximum time of two minutes to collect ticks from the cloth due to the large numbers of larvae present at some of the sites. We collected a representative sample of larvae into ethanol along the transect within a reasonable time using this method. We removed all larvae remaining on the cloth at the end of a transect using a lint roller. The larvae collected in vials were identified morphologically to the genus-level using taxonomic keys [7,42] and stored at -20°C. We counted the total number of larvae collected on lint rollers and extrapolated the total number belonging to each genus from the proportion of larvae collected into a vial from the identical transect and sampling session.

### Sample size determination for pathogen screening

To determine the number of individual *Amblyomma* larvae to extract per site and sampling session, we calculated sample size using the formula:

$$n=\frac{Z^{2}\times P\times (1-P)}{e^{2}}$$

where Z = value from standard normal distribution corresponding to desired confidence level, P = proportion of infected larvae, and e = desired precision or half desired confidence level width [43]. We used an estimated pathogen prevalence of 50% as a recent study from the same region found 19 out of 38 *A*. *hebraeum* nymphs were positive for *R*. *africae* [27]. This value is conservative because, for prevalence studies, the appropriate sample size is maximal when the true prevalence is 50% and decreases as the true prevalence approaches 0% or 100%. We selected the desired precision of 0.1 with a 95% confidence level. We determined that the appropriate sample size was n = 97 per site. To represent and replicate all land use types, we selected four sites in wildlife conservation reserves, two in cattle ranches, two in conservation ranches, and three in community lands, all sampled in the dry season of 2018, for pathogen screening. To quantify *R*. *africae* prevalence over time, we screened at least 97 individual *Amblyomma* larvae collected from Mbuluzi Game Reserve over each sampling session (dry seasons of 2017, 2018, 2019, and wet seasons of 2017/18 and 2018/19).

## Molecular analyses

### Primer and probe design

To determine the presence of *Rickettsia africae* in *Amblyomma hebraeum* larvae, we designed a multiplex qPCR assay to include *R*. *africae* specific primers and probe to target a 119 bp segment of the *ompB* gene [25], *A*. *hebraeum* specific primers and probe to target a 151 bp segment of the *CO1* gene, and Ixodidae tick primers and probe to target a 150 bp segment of the *16S* gene. We used the *16S* primers and probe as endogenous controls for each PCR reaction. The primer-probe combination specifically amplifying *R*. *africae* DNA was obtained from a previously published qPCR assay [25]. To design a primer-probe combination to detect *A*. *hebraeum* DNA, we generated potential primer-probe combinations using *CO1* gene sequence of *A*. *hebraeum* (KY457513). Candidate primer-probe sets were then evaluated for specificity to *A*. *hebraeum* and exclusion of co-occurring *Amblyomma* species using available *CO1* sequences (*Amblyomma exornatum*: MN150166; *Amblyomma marmoreum*: KY457516; *Amblyomma tholloni*: KT307493; *Amblyomma transversale*: MN150172; *Amblyomma variegatum*: GU062743). To design a primer-probe combination for use as an extraction control, we generated potential primer-probe combinations by using the *16S* gene of 14 tick species (S1 Table). The forward primer region was manually edited to include mixed nucleotides and enable detection of DNA from all hard tick genera tested. We generated all primer-probe combinations using the PrimerQuest Tool from Integrated DNA Technologies. We used the following criteria for identifying candidate primer-probe sets: optimal primer melting temperature of 61°C, optimal probe melting temperature of 68°C, and an amplicon size ranging from 100–150 bp.

### DNA extraction and pathogen detection

We performed all molecular analyses at the Molecular Ecology Laboratory at the University of Florida, FL, USA. We performed 1637 individual extractions of whole *Amblyomma* larvae using an adapted protocol from the Gentra Puregene Tissue Kit (Qiagen). Before extraction, larvae were rinsed in diH_2_O and 70% ethanol and dried. We made a single cut across the scutum using a sterile scalpel to preserve the tick exoskeleton for further morphological identification.

qPCR was performed in a 10 μL reaction volume using 2x QuantiTect Multiplex PCR Master Mix and 2 μL of template DNA (Table 1). All reaction plates included one positive control of *A*. *hebraeum* DNA, a three-point standard curve of *R*. *africae* plasmid positive controls at 10^3^, 10^5^, and 10^7^ copies per reaction, and one negative control (molecular grade water). We performed the real-time PCR-amplification assays using an Applied Biosystems QuantStudio 5 Real-Time PCR system (Thermo Fisher Scientific).

**Table 1:** The primers, probes, and thermocycling conditions^a^ that were used to detect *Rickettsia africae*, *Rickettsia*, and tick DNA in a novel multiplex qPCR assay for this study.

Target organism	Target gene	Primer name	Primer orientation	5’-3’ sequence	Reference	Concentration (nM)
Tick	*16S*	Tick-F2	Forward	CTCTAGGGATAACAGCGTWATAWT	This study	600
		Tick-R	Reverse	GTCTGAACTCAGATCAAGTAGG		600
		Tick-P1	Probe	6-FAM/TGCGACCTC/ZEN/GATGTTGGATTAGGA/3’IowaBlack		250
*Amblyomma hebraeum*	*CO1*	Aheb_F	Forward	CATCATAATTGGCGGGTTTG	This study	400
		Aheb_R	Reverse	AGTAAACAAAGAGATGGTGGTA		400
		Aheb_P	Probe	Cy5/TGACTAGTT/TAO/CCAATTATGCTAGGTGCCC/3’IowaBlack		250
*Rickettsia africae*	*ompB*	Raf1797F	Forward	TTGGAGCTAATAATAAAACTCTTGGAC	[25]	100
		Raf1915R	Reverse	GAATTGTACTGCACCGTTATTTCC		100
		Raf1879P	Probe	HEX/CGCGATGTTAATAGCAACATCACC GCCACTATCGCG/Black Hole Quencher		250

^a^Thermocycling conditions were as follows: initial denaturation of 95°C for 15 minutes followed by 40 cycles of 95°C for 45 seconds and 60°C for 90 seconds.

To evaluate the specificity of the *R*. *africae* primer-probe set, we screened DNA from previously identified samples of *R*. *africae*, *Rickettsia amblyommatis*, *Rickettsia belli*, Candidatus *Rickettsia barbariae*, *Rickettsia conorii*, *Rickettsia massiliae*, *Rickettsia parkeri*, and *Rickettsia rhipicephali*. Furthermore, we used conventional PCR (cPCR) to amplify the *ompB* gene of 18 randomly selected positive samples as previously described [27]. To evaluate the specificity of the *A*. *hebraeum* primer-probe set, we screened DNA from previously identified ticks, including five species of *Amblyomma* (*Amblyomma americanum*, *A*. *hebraeum*, *Amblyomma maculatum*, *Amblyomma marmoreum*, and *Amblyomma rotumdatum*), *Dermacentor variabilis*, *Haemaphysalis elliptica*, *Ixodes scapularis*, and six species of *Rhipicephalus* (*Rhipicephalus appendiculatus*, *Rhipicephalus decoloratus*, *Rhipicephalus maculatus*, *Rhipicephalus microplus*, *Rhipicephalus sanguineus*, *Rhipicephalus simus*). We used cPCR to amplify the *CO1* gene of 13 randomly selected individuals positive for the *A*. *hebraeum* probe and seven randomly selected individuals negative for the *A*. *hebraeum* probe using previously described methods [27]. To further characterize the *Amblyomma* larvae negative for the *A*. *hebraeum* probe, we used cPCR to amplify the *12S* gene and *ITS2* gene of two randomly selected individuals using previously described methods [27]. All cPCR amplicons were purified using Exo-SAP (New England Biolabs) and sent for sequencing with the same primers (Eurofins Genomics). We assembled sequences with Geneious Prime 2021.1.1.

To evaluate the sensitivity of the detection of *R*. *africae* in the qPCR assay, we obtained a synthetic gene strand spanning the *R*. *africae ompB* target region (Eurofins Genomics). We used the restriction enzyme EcoRI (New England BioLabs) to linearize the plasmid. Ten-fold serial dilutions ranging from 1 x 10^7^ copies to 1 copy *R*. *africae ompB* DNA in triplicate were amplified using the previously mentioned qPCR conditions. We identified the detection limit as the lowest concentration of DNA with detectable fluorescence above the background prior to cycle 40. We calculated goodness of fit (R^2^) for the standard curve and reaction efficiency (E) based on the slope of the standard curve.

## Data analyses

### Density of larvae

To calculate the density of questing *Amblyomma* larvae (hereafter: DOL) per 100m^2^ for each site, we divided the number of collected *Amblyomma* larvae on a transect by the total distance sampled in m^2^ and multiplied by 100. The mean and variance of DOL were then calculated at the site level using the transect-level densities. To assess the influence of the number of unique transects on DOL, we evaluated the association between the number of unique transects per site and the mean DOL and variance of DOL using the Kruskal-Wallis test.

To investigate the relationship between DOL with a categorical variable representing land use (conservation reserve, cattle ranch, conservation ranch, community land), we used the DOL for each site collected during the dry season of 2018. We used negative binomial generalized linear mixed models to account for overdispersion in the count data. We modeled log-transformed DOL with land use as a fixed variable and location (i.e., name of the specific reserve, as more than one sampling site in some conservation areas) as a random variable. We checked model fit by simulating the residuals from the model and quantifying deviation. The null model and land use model were ranked using Akaike Information Criterion (AIC) and compared by computing the analysis of variance.

We obtained monthly precipitation data from the Eswatini Meteorological Services weather station located within the study area (Tabankulu) from 2016 to 2019. We calculated the total wet season (October to March) and the total dry season (April to September) precipitation for each year over the four years. To test for seasonal variability (wet vs. dry) in DOL, we conducted a two-sample t-test. Then, we used a simple linear regression to test if the current season total precipitation, the total precipitation 12 months prior, or the total precipitation 24 months prior significantly predicted DOL per sampling session in Mbuluzi Game Reserve.

### Prevalence of *R*. *africae* and density of infected larvae

We removed all individual larval samples with a cycling threshold (C_T_) > 40 for the tick endogenous control from further analysis. To calculate the infection prevalence (hereafter: LIP) of *A*. *hebraeum* larvae with *R*. *africae*, we included only samples with a C_T_ < 38 for the *A*. *hebraeum* qPCR probe. LIPs with 95% exact binomial confidence intervals (95%CI) were calculated at the site-level using Fisher’s exact test [44]. Using the Kruskal-Wallis test, we evaluated the association between the number of transects from which *A*. *hebraeum* larvae were collected per site and LIP. We also used a simple linear regression to test if DOL significantly predicted LIP.

We calculated the density of infected larvae (hereafter: DIL) by multiplying the DOL and LIP for each site. We used binomial and negative binomial generalized linear models to investigate the relationship of both LIP and DIL with land use. Model fit was checked by simulating the residuals from the model and quantifying deviation. The null model and land use model were ranked using AIC. To test for seasonal variability (wet vs. dry) in LIP and DIL, we conducted a two-sample t-test. We used a simple linear regression to test if the current season total precipitation, the total precipitation six months prior, or the total precipitation 12 months prior significantly predicted LIP and DIL. We performed all statistical analyses using the R software version 4.0.5 [45] and implemented models using the package GLMMadpative [46].

## Results

### Density of larvae

**Fig 1:**
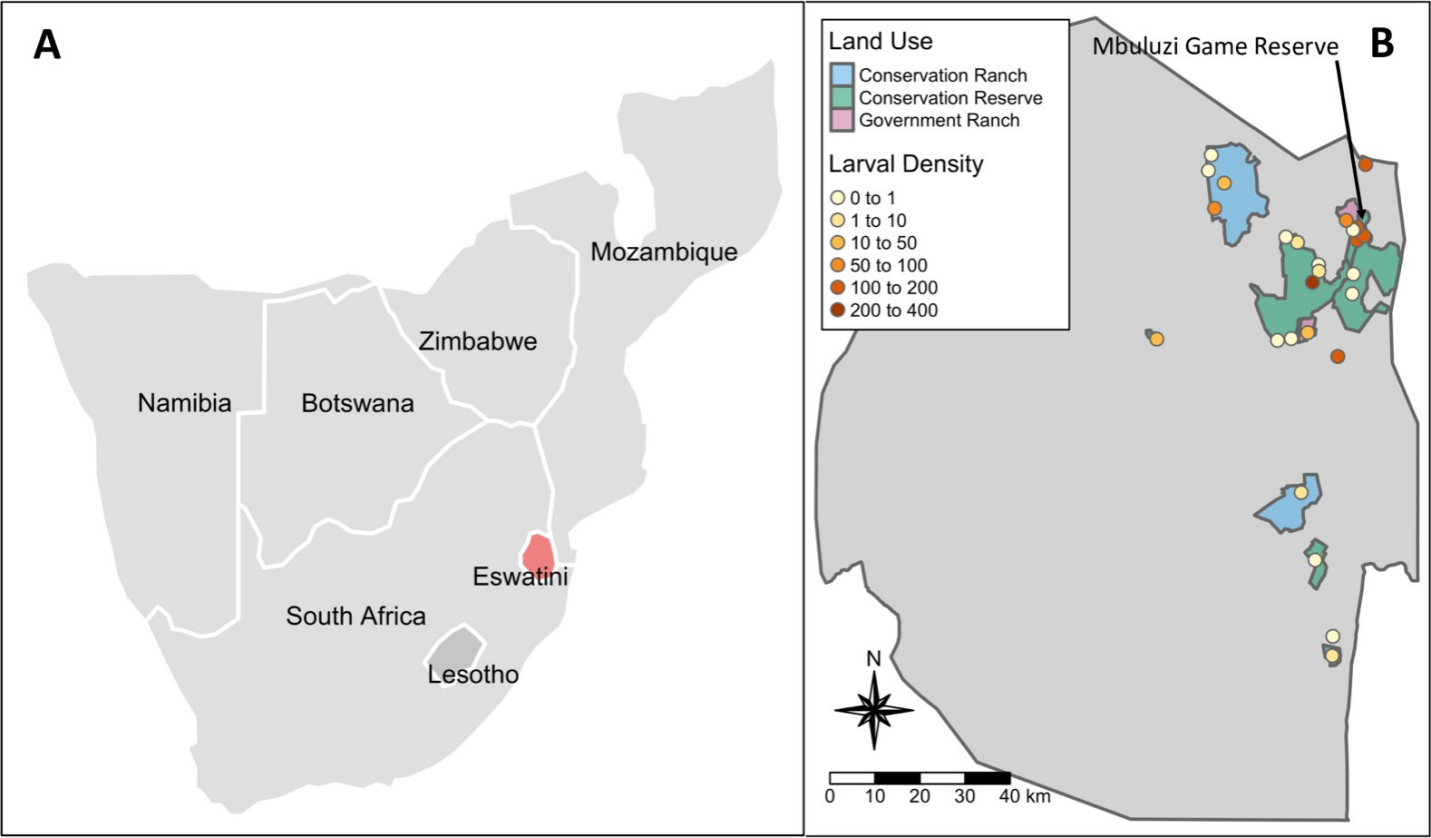
Study area. (A) Map of southern Africa with Eswatini indicated in red and (B) map of Eswatini indicating land use and the density of *Amblyomma* larvae per 100m^2^ at sampling sites during the dry season of 2018. Sampling sites not located within a conservation reserve, cattle ranch, or conservation ranch were in community lands. Made with Natural Earth (https://www.naturalearthdata.com.

We collected 12,711 *Amblyomma* larvae across 19 of the 26 sites sampled during the dry season of 2018. We determined the apparent proportion of *A*. *hebraeum* larvae out of all *Amblyomma* larvae based on a subset of our dataset set to be 97%. No *Amblyomma* larvae were detected in seven sites. The average DOL across all sites during the dry season of 2018 was 51 ± 16 individuals per 100m^2^ (Fig 1). The highest average DOL from a single site was 333 individuals per 100m^2^ in Hlane Royal National Park conservation reserve (S2 Table). We observed no significant difference in DOL among land use categories, as the null model outperformed the model including land use (null model AIC = 111.0; LU model AIC = 114.7; anova *p*-value = 0.5) (S3 Table). Neither the mean nor variance of *Amblyomma* larval density were significantly associated with the number unique transects sampled per site (mean DOL: H(5) = 3.6, *p* = 0.6; variance DOL: H(5) = 3.9, *p* = 0.6).

DOL varied seasonally and annually over the five sampling sessions spanning three dry seasons and two wet seasons in Mbuluzi Game Reserve. We observed higher DOL in the wet seasons than dry seasons, but this difference was not statistically significant (mean dry = 73 ± 23 per 100m^2^; mean wet = 182 ± 90 per 100m^2^; t-test: t(4) = -1.2; p = 0.43). The wet season of 2015/16 received approximately 62% less rainfall than an average wet season. The simple linear regression of the mean precipitation 24 months prior and DOL was statistically significant (ß = 0.74; R^2^ = 0.88; F(1,3) = 21.5; p = 0.02) (S4 Table). The high DOL in the wet season of 2018/19 drove this relationship. Neither the linear regression testing precipitation 12 months prior nor current precipitation significantly predicted DOL, but both showed positive relationships with wetter years resulting in higher DOL.

### qPCR assay validation

The tick primer-probe set successfully amplified DNA from all tick species tested. The *A*. *hebraeum* primer-probe set only amplified DNA from *A*. *hebraeum*. We observed no amplification for any other tick species, including the other species of *Amblyomma*. The *R*. *africae* specific primer-probe set amplified *R*. *africae* DNA, but not any other species of *Rickettsia* DNA tested. The limit of detection for *R*. *africae* primer-probe set in the multiplex assay was 1000 copies of the linearized DNA plasmid. The standard curve had a goodness of fit of R^2^ = 0.998 and reaction efficiency of E = 58.5%.

### Molecular identification of *Amblyomma* larvae

1558 out of 1605 *Amblyomma* larvae included in molecular analyses were identified as *A*. *hebraeum* using the multiplex qPCR assay. We validated the identification of 13 randomly selected *A*. *hebraeum* individuals by sequencing the *CO1* gene and finding 100% similarity (644 out of 644 base pairs) to *A*. *hebraeum* [KY457513]. From the subset of *Amblyomma* larvae that did not fluoresce the *A*. *hebraeum* probe, we sequenced the *CO1* gene from 7 randomly selected individuals and found all identical and had a 95.2% similarity (613 out of 644 base pairs) to *Amblyomma marmoreum* [KY457515]. The *12S* and *ITS2* genes from two individuals of *Amblyomma* larvae had 96.5% similarity (360 out of 373 base pairs) to *A*. *marmoreum* [KY457515] and 99.9% similarity (1023 out of 1024 base pairs) to *A*. *marmoreum* [KY457491], respectively. We archived our *Amblyomma marmoreum* sequences to GenBank under the accession numbers: OM315185 (*CO1*), OM320577 (*12S*), and OM320578 (*ITS2*).

### *R*. *africae* infection prevalence in *Amblyomma* larvae

We quantified LIP at 11 sites and found a study-wide infection prevalence of 64.9% (95% CI: 62.1–67.6%) for *R*. *africae* in *A*. *hebraeum* larvae. The highest LIP from a single site was 99% (95% CI: 94.6–100%) in a conservation ranch, and the lowest LIP was 1% (95% CI: 0–5.5%) in community lands near the rural settlement of Lomahasha (S2 Table). Across all sites, there was no significant relationship between DOL and LIP (R^2^ = 0.22, F(1,9) = 2.6, *p* = 0.14) and LIP was not significantly associated with land use as the null model outperformed the model including land use (null model AIC = 111.8; LU model AIC = 123.8) (S5 Table). Neither the mean nor variance of LIP were significantly associated with the number of unique transects from which we collected and tested larvae from each plot (mean LIP: H(7) = 4.9, *p* = 0.7; variance LIP: H(6) = 5.9, *p* = 0.4).

In Mbuluzi Game Reserve, LIP ranged from 51.6% (95% CI: 44.8–58.3%) during the dry season of 2018 to 90.5% (95% CI: 82.8–95.6%) in the wet season of 2018/19 (S6 Table). Overall, we observed no significant difference in LIP between the two seasons (mean dry = 73.8% ± 11.3%; mean wet = 73.6% ± 17.0%; t-test: t(4) = 0.01; p = 0.99). We identified one out of 46 *A*. *marmoreum* larvae to be positive for *R*. *africae* (both tick and *R*. *africae* identification confirmed by conventional PCR and sequencing) for a prevalence of 2.2% (95% CI: 0.1–11.5%).

To further validate the specificity of our assay, we submitted 18 randomly selected samples positive for *R*. *africae* for Sanger sequencing using the conventional genus-wide *Rickettsia ompB* PCR assay. 17 of the 18 samples [OL505022] had 100% similarity (829 out of 829 base pairs) to *R*. *africae* [CP001612], and one sample [OL505023] with two base pairs differences had 99.8% similarity to *R*. *africae* [CP001612].

### Density of infected larvae

**Fig 2:**
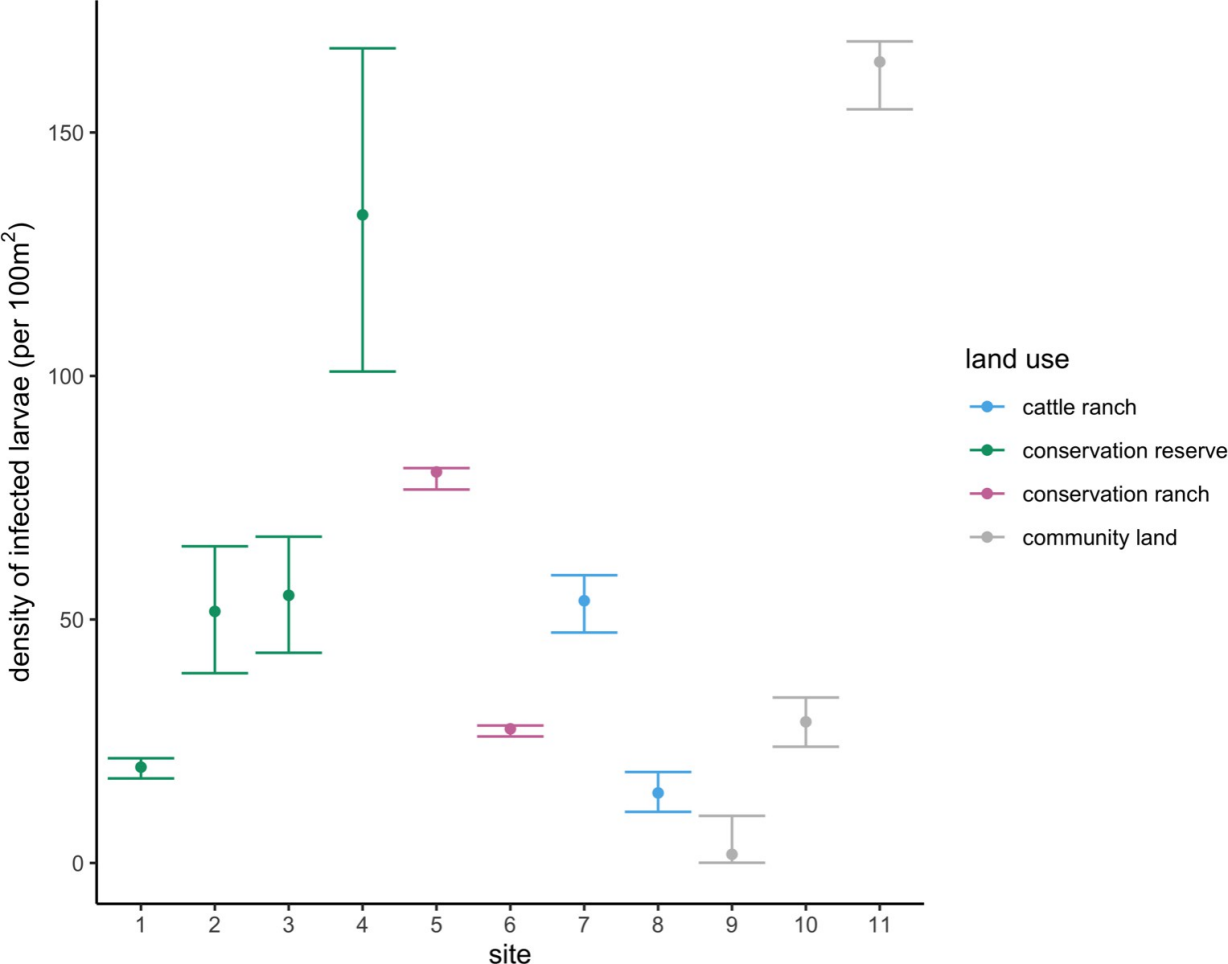
Density of *Amblyomma hebraeum* larvae infected with *Rickettsia africae* at the 11 sampling sites surveyed in the dry season of 2018, from which we estimated infection prevalence. There was no significant difference by land use category.

The average DIL across all sites during the 2018 dry season was 57.3 (95% CI: 49–65) individuals per 100m^2^ (S2 Table). The highest DIL from a single site was 165 (95% CI: 154–169) individuals per 100m^2^ in community lands near the rural settlement Sitsatsaweni (Fig 2). DIL was not significantly associated with land use as the null model outperformed the model that included land use (null AIC = 42.5; LU model AIC = 48.1) (S7 Table). In Mbuluzi Game Reserve, DIL ranged from 31.3 (95% CI: 28–33) individuals per 100m^2^ during the 2019 dry season to 246 (95% CI: 225–260) individuals per 100m^2^ during the 2018/19 wet season (S6 Table). We observed higher DIL in the wet seasons than in the dry seasons, but this difference was not statistically significant (mean dry = 48.4 ± 8.7 per 100m^2^; mean wet = 149 ± 97.2 per 100m^2^; t-test: t(4) = -1.0; p = 0.49). The simple linear regression testing if the mean precipitation 24 months prior predicted DIL was statistically significant (ß = 0.71; R^2^ = 0.88; F(1,3) = 21.7; p = 0.02) (S4 Table). Neither the linear regression testing precipitation 12 months prior nor current precipitation significantly predicted DIL, but both showed positive relationships with wetter years resulting in higher DIL.

## Discussion

This study detected large numbers of *A*. *hebraeum* larvae and a high prevalence of *R*. *africae* DNA in *A*. *hebraeum* larval ticks collected in Eswatini. We demonstrated that *A*. *hebraeum* larvae are abundant, ubiquitous ticks and act as the primary vector of *R*. *africae* in southern Africa. These results illustrate that these aggressive host-seeking larvae are widespread in the lowveld savanna of Eswatini and, due to the high infection rate, likely play a significant role in human infection with *R*. *africae*.

We observed a large variance in the density of *Amblyomma* larvae across our study due to the patchy distribution of larval ticks across space. Despite this variability, we found ticks in all land use types (conservation areas, ranches, and community lands) with high densities of *Amblyomma* larvae infected with *R*. *africae*. The high densities of infected ticks suggest significant entomological risk of ATBF regardless of land use. The variability of the density of infected larvae was driven by the variability in the density of larvae, as larval infection prevalence was high over both space and time. *Amblyomma hebraeum* represented the vast majority (~95%) of our *Amblyomma* larvae included in molecular analysis. Overall, the high contact rate with infected larval *A*. *hebraeum* reported in this study shows that *R*. *africae* transmission to humans may be an undiagnosed public health issue in Eswatini.

Apart from traveler cases, there are limited studies on the epidemiology of *R*. *africae* infections. To date, there are no published records describing disease incidence of ATBF or seroprevalence of *R*. *africae* in local populations from Eswatini. However, the seroprevalence of past infection with SFG *Rickettsia* in people from a rural area in South Africa, approximately 100 km away from Eswatini, was 63–92% [13]. While the morbidity and mortality caused by *R*. *africae* in communities across sub-Saharan Africa are unknown, cases of SFG *Rickettsia* can be fatal [47]. Elsewhere in sub-Saharan Africa, a study from Tanzania found 57 out of 641 (8.9%) patients with fever and paired serology had *R*. *africae* infection [9]. Rickettsial infections are a reality in sub-Saharan Africa, but the limited surveillance for nonmalarial febrile illnesses severely limits our understanding of the epidemiology and impact of diseases caused by *R*. *africae* and other SFG *Rickettsia*.

The prevalence of *R*. *africae* in *Amblyomma* ticks is consistent with previous studies from southern Africa. While most of these studies only tested the infection rate for the adult life stage, our prevalence range of 52–90% in larvae is comparable to that found in adults (60–80%) [24,28–30,48]. This result provides evidence that the proportion of infected individuals remains relatively constant over both life stages and generation, suggesting efficient transstadial and transovarial transmission in nature. The larval sampling and calculation of larval infection prevalence across time were limited to sites within a single conservation area. While this sampling scheme limits the interpretation of our data, we provide empirical evidence that infected *A*. *hebraeum* ticks are searching for hosts during both the wet and dry seasons, indicating a continuous risk of transmission throughout the year in this study region. Ultimately, this result supports the role of *A*. *hebraeum* ticks as the main reservoir of *R*. *africae* in southern Africa.

For this study, we selected sites in savanna grasses where ticks could be present in the vegetation, and human activity could result in exposure to ticks. While we tested an adequate number of individual samples for each site to achieve a 95% confidence interval within +/- 0.1 of the prevalence estimate, more sampling locations may have provided additional power to differentiate the density of larvae or the infection prevalence of larvae between land use types. Despite these limitations, we found large numbers of *A*. *hebraeum* larvae and a high infection prevalence of *R*. *africae* across the study area and duration.

The sampling associated with this study found seven different tick species collected as adults or nymphs [27]. Tick species richness was greatest in wildlife conservation areas (n = 7) and lowest in cattle-only ranches and community lands (n = 4). Tick-borne pathogen testing of adults and nymphs detected *Anaplasma*, *Ehrlichia*, *Rickettsia*, *Babesia*, *Heptatozoon*, and *Theileria* DNA. Detected *Rickettsia* species included three described species (*R*. *africae*, *Rickettsia conorii*, and *Rickettsia massiliae*), one candidate species (Ca. *Rickettsia barbariae*), and three undescribed genotypes of *Rickettsia*. *Rickettsia africae* was detected in *A*. *hebraeum*, with 19/38 (50%) of nymphs and 1/12 (8.3%) of adults testing positive [27].

The novel multiplex qPCR assay designed in this study demonstrated high specificity as there was no cross-reactivity of the *R*. *africae* primer-probe set with other species of *Rickettsia*. The *A*. *hebraeum* primer-probe set also did not cross-react with other tick species. The sensitivity and reaction efficiency of the assay fell below desired levels for diagnostic assays [49]. The less-than-ideal sensitivity of the assay may have resulted in false negatives in this study. The low sensitivity means that even more than the detected 65% of *A*. *hebraeum* larvae may indeed be infected with *R*. *africae* and further increase the risk of transmission. Nonetheless, the assay was able to identify the high prevalence of *R*. *africae* in our samples.

This study’s collection included the second published record of *A*. *marmoreum* in Eswatini and the first *A*. *marmoreum* larvae from the vegetation. These samples also included the first documentation of *R*. *africae* infection from *A*. *marmoreum*. While *A*. *marmoreum* has a lower infection prevalence of *R*. *africae* than *A*. *hebraeum*, our results show that larval *A*. *marmoreum* could play a role as a vector of ATBF as larvae are known to attach to humans [21] and can be abundant in the vegetation [50].

Previous studies found the peak of *A*. *hebraeum* larval activity in vegetation in the Eastern Cape to the south of Eswatini and Kruger National Park, South Africa to the north of Eswatini is highest during the wet season (November to January) and is lowest during the dry season (June to October) [51,52]. While our study did find the density of *Amblyomma* larvae to be highest during the wet season, the regional drought that affected the region in 2015 and 2016 may have influenced the relationship. A long-term (~13 years) study on the density of questing ticks also observed lower numbers of questing *A*. *hebraeum* larvae in the years immediately following a drought [51]. Our tick density and rainfall observations 24 months before sampling were strongly correlated, while current rainfall and rainfall 12 months prior were only weakly correlated. This relationship suggests that there may be a multi-year lag in tick population recovery after extreme weather events. Furthermore, seasonal rainfall may not be the primary determinant of questing ticks, and climate-induced variation in host populations may also have influenced the observed relationship. Over time, the variability in tick numbers may reflect the effect of climatic conditions on free-living ticks and their hosts.

## Conclusion

The findings from this study add further evidence that *A*. *hebraeum* is an abundant tick species, and the high percentage of infected larval ticks with *R*. *africae* increases the probability an infected tick will bite humans. This work, along with the documentation of high seroprevalence in humans [10–14], adds to the evidence that rickettsiosis contributes to febrile bacterial disease in Africa and the impact of its neglect on patient health globally is potentially substantial. There is a need to increase awareness of the importance of the larval life stage of *A*. *hebraeum* in the transmission of ATBF to people in southern Africa, including tourists who engage in outdoor activities, locals who work in conservation areas and cattle ranches, and local people who live in rural settings with either domestic or wild animals near their home. Overall, rickettsioses, specifically African tick-bite fever, warrant increased recognition by policymakers, funders, healthcare workers, and scientists.

## Supporting information

S1 Table Organism name and Genbank accession number of tick sequences used to develop the qPCR assay. (DOCX)

S2 Table Summary of *A*. *hebraeum* larvae by sampling site. For land use, wildlife refers to wildlife conservation areas, and mixed refers to conservation lands with both cattle and wildlife. Mean DOL = mean density of larvae per 100m^2^. No. tested = the number of individual larvae screened for *R*. *africae* using the multiplex qPCR. No. positive = the number of individual larvae positive for *R*. *africae*. LIP = larval infection prevalence. DIL = density of infected larvae per 100m^2^.
(DOCX)

S3 Table Null and land use model outputs with the density of *Amblyomma* larvae (DOL) as the response variable using a negative binomial GLMM. (DOCX)

S4 Table Linear model outputs comparing the density of *Amblyomma* larvae (DOL) and the density of infected *Amblyomma* larvae (DIL) to precipitation. (DOCX)

S5 Table Null and land use model outputs with *A*. *hebraeum* larval infection prevalence (LIP) as the response variable using a binomial GLM. (DOCX)

S6 Table Summary of the density of *Amblyomma* larvae per 100m^2^ (DOL), larval infection prevalence with *Rickettsia africae* (LIP), and density of *Amblyomma* larvae infected with *R*. *africae* per 100m^2^ (DIL) in Mbuluzi Game Reserve over five sampling sessions. (DOCX)

S7 Table Null and land use model outputs with the density of *A*. *hebraeum* larvae infected with *R*. *africae* (DIL) as the response variable using a negative binomial GLM. (DOCX)
