# Supplementary material for: Entomological risk of African tick-bite fever (Rickettsia africae infection) in Eswatini
Source: PLoS Negl Trop Dis. 2022 May 16;16(5):e0010437. doi: 10.1371/journal.pntd.0010437 (PMC9135330; doi:10.1371/journal.pntd.0010437)
Supplement: S1 Table — (DOCX) [file pntd.0010437.s001.docx]

S1 Table. Organism name and Genbank accession number of tick sequences used to develop the qPCR assay.

| Organism | Accession # |
| --- | --- |
| *Ixodes ricinus* | L34292 |
| *Ixodes scapulars* | L34293 |
| *Ixodes pacificus* | L34296 |
| *Dermacentor andersoni* | L34299 |
| *Dermacentor variabilis* | L34300 |
| *Rhipicephalus appendiculatus* | L34301 |
| *Rhipicephalus sanguineus* | L34302 |
| *Rhipicephalus turanicus* | L34303 |
| *Rhipicephalus microplus* | L34310 |
| *Amblyomma variegatum* | L34312 |
| *Amblyomma americanum* | L34313 |
| *Amblyomma hebraeum* | L34316 |
| *Amblyomma cajennense* | L34317 |
| *Amblyomma maculatum* | L34318 |
