# Supplementary material for: Entomological risk of African tick-bite fever (Rickettsia africae infection) in Eswatini
Source: PLoS Negl Trop Dis. 2022 May 16;16(5):e0010437. doi: 10.1371/journal.pntd.0010437 (PMC9135330; doi:10.1371/journal.pntd.0010437)
Supplement: S2 Table — For land use, wildlife refers to wildlife conservation areas, and mixed refers to conservation lands with both cattle and wildlife. Mean DOL = mean density of larvae per 100m2. No. tested = the number of individual larvae screened for R. africae using the multiplex qPCR. No. positive = the number of individual larvae positive for R. africae. LIP = larval infection prevalence. DIL = density of infected larvae per 100m2. (DOCX) [file pntd.0010437.s002.docx]

S2 Table. Summary of *A. hebraeum* larvae by sampling site. For land use, wildlife refers to wildlife conservation areas, and mixed refers to conservation lands with both cattle and wildlife. Mean DOL = mean density of larvae per 100m^2^. No. tested = the number of individual larvae screened for *R. africae* using the multiplex qPCR. No. positive = the number of individual larvae positive for *R. africae*. LIP = larval infection prevalence. DIL = density of infected larvae per 100m^2^.

| **Site ID** | **Location** | **Land use** | **longitude** | **latitude** | **mean DOL** | **no. tested** | **no. positive** | **LIP estimate** | **LIP 95% CI** | **DIL estimate** | **DIL 95% CI** |
| --- | --- | --- | --- | --- | --- | --- | --- | --- | --- | --- | --- |
| NK | Nkalashane | cattle ranch | 31.974 | -26.12169 | 68.17 | 100 | 80 | 80.0 | 70.8 - 87.3 | 54.5 | 48.3 - 59.5 |
| RIC | Ndukuyamangendla | cattle ranch | 31.88825 | -26.34553 | 42.33 | 100 | 34 | 34.0 | 24.8 - 44.2 | 14.4 | 10.5 - 18.7 |
| LO1 | Lomahasha | communal | 32.01728 | -26.01039 | 176.00 | 99 | 1 | 1.0 | 0.03 - 5.5 | 1.8 | 0.05 - 9.7 |
| MA1 | Maphiveni | communal | 31.98955 | -26.141 | 53.20 | 114 | 62 | 54.4 | 44.8 - 63.7 | 28.9 | 23.8 - 33.9 |
| SE | Nisela | communal | 31.94426 | -26.95095 | 0.00 | NA | NA | NA | NA | NA | NA |
| SI | Sitsatsaweni | communal | 31.95534 | -26.39316 | 169.83 | 96 | 93 | 96.9 | 91.1 - 99.4 | 164.5 | 154.8 - 168.7 |
| DO | Dombeya | wildlife | 31.55131 | -26.35841 | 24.40 | 93 | 75 | 80.6 | 71.1 - 88.1 | 19.7 | 17.4 - 21.5 |
| HH1 | Hlane | wildlife | 31.8653 | -26.16586 | 3.67 | NA | NA | NA | NA | NA | NA |
| HH2 | Hlane | wildlife | 31.83953 | -26.15501 | 0.00 | NA | NA | NA | NA | NA | NA |
| HH3 | Hlane | wildlife | 31.91228 | -26.21034 | 0.00 | NA | NA | NA | NA | NA | NA |
| HL1 | Mbuluzi | wildlife | 31.99862 | -26.1614 | 123.67 | 91 | 38 | 41.8 | 31.5 - 52.5 | 51.6 | 39.0 - 65.0 |
| HL2 | Mbuluzi | wildlife | 32.01388 | -26.15297 | 103.83 | NA | NA | NA | NA | NA | NA |
| HM1 | Hlane | wildlife | 31.91316 | -26.22316 | 1.25 | NA | NA | NA | NA | NA | NA |
| HM2 | Mbuluzi | wildlife | 31.99833 | -26.13594 | 118.08 | 101 | 47 | 46.5 | 36.5 - 56.7 | 54.9 | 43.2 - 67.0 |
| LL1 | Hlane | wildlife | 31.89875 | -26.24525 | 332.67 | 100 | 40 | 40.0 | 30.3 - 50.3 | 133.1 | 100.9 - 167.3 |
| LL2 | Mlawula | wildlife | 31.99002 | -26.22884 | 0.42 | NA | NA | NA | NA | NA | NA |
| MH | Mhlosinga | wildlife | 31.90481 | -26.79881 | 0.70 | NA | NA | NA | NA | NA | NA |
| ML1 | Hlane | wildlife | 31.82066 | -26.36108 | 0.17 | NA | NA | NA | NA | NA | NA |
| ML2 | Mlawula | wildlife | 31.98754 | -26.26839 | 0.33 | NA | NA | NA | NA | NA | NA |
| MM1 | Hlane | wildlife | 31.85142 | -26.35743 | 0.00 | NA | NA | NA | NA | NA | NA |
| NI | Nisela Reserve | wildlife | 31.94298 | -26.98956 | 4.67 | NA | NA | NA | NA | NA | NA |
| BU2 | Bushlands | mixed | 31.8736 | -26.66427 | 2.67 | NA | NA | NA | NA | NA | NA |
| LH1 | IYSIS | mixed | 31.68042 | -26.09809 | 81.08 | 100 | 99 | 99.0 | 94.6 - 100.0 | 80.3 | 76.7 - 81.1 |
| LH2 | IYSIS | mixed | 31.66662 | -26.02253 | 0.00 | NA | NA | NA | NA | NA | NA |
| LM1 | IYSIS | mixed | 31.70196 | -26.04761 | 28.42 | 100 | 97 | 97.0 | 91.5 - 99.4 | 27.6 | 26.0 - 28.2 |
| LM2 | IYSIS | mixed | 31.67312 | -25.99159 | 0.00 | NA | NA | NA | NA | NA | NA |
