# Supplementary material for: Entomological risk of African tick-bite fever (Rickettsia africae infection) in Eswatini
Source: PLoS Negl Trop Dis. 2022 May 16;16(5):e0010437. doi: 10.1371/journal.pntd.0010437 (PMC9135330; doi:10.1371/journal.pntd.0010437)
Supplement: S3 Table — (DOCX) [file pntd.0010437.s003.docx]

S3 Table. Null and land use model outputs with the density of *Amblyomma* larvae (DOL) as the response variable using a negative binomial GLMM. ** < 0.01; * p < 0.05.

|  | **Intercept** | **LU:**  **communal** | **LU:**  **conservation** | **LU:**  **mixed** | **AIC** |
| --- | --- | --- | --- | --- | --- |
| DOL | 0.840** (0.218) |  |  |  | 110.0 |
| DOL | 1.392** (0.624) | -0.125 (0.772) | -0.762 (0.690) | -0.802 (0.795) | 114.7 |
