# Supplementary material for: Entomological risk of African tick-bite fever (Rickettsia africae infection) in Eswatini
Source: PLoS Negl Trop Dis. 2022 May 16;16(5):e0010437. doi: 10.1371/journal.pntd.0010437 (PMC9135330; doi:10.1371/journal.pntd.0010437)
Supplement: S4 Table — (DOCX) [file pntd.0010437.s004.docx]

S4 Table. Linear model outputs comparing the density of *Amblyomma* larvae (DOL) and the density of infected *Amblyomma* larvae (DIL) to precipitation.

| **Response Variable** | **Precipitation** | **Intercept** | **Precipitation** | **Adjusted R2** |
| --- | --- | --- | --- | --- |
| DOL | current | 40.48 (49.71) | 0.282 (0.143) | 0.42 |
| DOL | 12 months prior | 22.77 (51.02) | 0.399 (0.179) | 0.50 |
| DOL | 24 months prior | 1.36 (29.86) | 0.740* (0.158) | 0.84 |
| DIL | current | 19.70 (51.28) | 0.256 (0.148) | 0.34 |
| DIL | 12 months prior | -2.83 (48.04) | 0.390 (0.168) | 0.52 |
| DIL | 24 months prior | -22.29 (28.69) | 0.710* (0.152) | 0.84 |
