# Supplementary material for: Entomological risk of African tick-bite fever (Rickettsia africae infection) in Eswatini
Source: PLoS Negl Trop Dis. 2022 May 16;16(5):e0010437. doi: 10.1371/journal.pntd.0010437 (PMC9135330; doi:10.1371/journal.pntd.0010437)
Supplement: S5 Table — (DOCX) [file pntd.0010437.s005.docx]

S5 Table. Null and land use model outputs with *A. hebraeum* larval infection prevalence (LIP) as the response variable using a binomial GLM. ** < 0.01; * p < 0.05.

|  | Intercept | LU:  communal | LU:  conservation | LU:  mixed | AIC |
| --- | --- | --- | --- | --- | --- |
| LIP | 61.0** (9.6) |  |  |  | 110.3 |
| LIP | 57.0 (22.0) | -6.24  (28.4) | -4.77  (26.94) | 41.00 (31.11) | 111.8 |
