# Supplementary material for: Entomological risk of African tick-bite fever (Rickettsia africae infection) in Eswatini
Source: PLoS Negl Trop Dis. 2022 May 16;16(5):e0010437. doi: 10.1371/journal.pntd.0010437 (PMC9135330; doi:10.1371/journal.pntd.0010437)
Supplement: S6 Table — (DOCX) [file pntd.0010437.s006.docx]

S6 Table. Summary of the density of *Amblyomma* larvae per 100m^2^ (DOL), larval infection prevalence with *R. africae* (LIP), and density of *Amblyomma* larvae infected with *R. africae* per 100m^2^ (DIL) in Mbuluzi Game Reserve over 5 sampling sessions.

| **Year** | **Season** | **mean DOL** | **mean LIP** | **LIP 95% CI** | **mean DIL** | **DIL 95% CI** |
| --- | --- | --- | --- | --- | --- | --- |
| 2017 | dry | 67.31 | 81 | 71.9-88.2 | 54.52 | 48.4-59.4 |
| 2017 | wet | 91.89 | 56.6 | 46.2-66.5 | 52.01 | 42.5-61.1 |
| 2018 | dry | 115.22 | 51.6 | 44.8-58.3 | 59.45 | 51.6-67.2 |
| 2018 | wet | 272.25 | 90.5 | 82.8-95.6 | 246.39 | 225.4-260.3 |
| 2019 | dry | 35.25 | 88.8 | 80.8-94.3 | 31.30 | 28.5-33.2 |
