# Supplementary material for: Entomological risk of African tick-bite fever (Rickettsia africae infection) in Eswatini
Source: PLoS Negl Trop Dis. 2022 May 16;16(5):e0010437. doi: 10.1371/journal.pntd.0010437 (PMC9135330; doi:10.1371/journal.pntd.0010437)
Supplement: S7 Table — (DOCX) [file pntd.0010437.s007.docx]

S7 Table. Null and land use model outputs with the density of *A. hebraeum* larvae infected with *R. africae* (DIL) as the response variable using a negative binomial GLM. ** < 0.01; * p < 0.05.

|  | Intercept | LU:  communal | LU:  conservation | LU:  mixed | AIC |
| --- | --- | --- | --- | --- | --- |
| DIL | 1.289** (0.158) |  |  |  | 42.5 |
| DIL | 1.215** (0.385) | 0.166 (0.460) | 0.140 (0.527) | -0.059 (0.503) | 48.1 |
